# Expression profile and functional role of S100A14 in human cancer

**DOI:** 10.18632/oncotarget.26861

**Published:** 2019-04-26

**Authors:** Suyog Basnet, Sunita Sharma, Daniela Elena Costea, Dipak Sapkota

**Affiliations:** ^1^ Department of BioSciences, Faculty of Mathematics and Natural Sciences, University of Oslo, Oslo, Norway; ^2^ Department of Clinical Dentistry, Centre for Clinical Dental Research, University of Bergen, Bergen, Norway; ^3^ Gade Laboratory for Pathology, Department of Clinical Medicine, Faculty of Medicine and Dentistry, University of Bergen, Bergen, Norway; ^4^ Centre for Cancer Biomarkers (CCBIO), Faculty of Medicine and Dentistry, University of Bergen, Bergen, Norway; ^5^ Department of Pathology, Haukeland University Hospital, Bergen, Norway; ^6^ Department of Oral Biology, Faculty of Dentistry, University of Oslo, Oslo, Norway

**Keywords:** S100 proteins, cancer, differentiation, expression, prognosis

## Abstract

S100A14 is one of the new members of the multi-functional S100 protein family. Expression of S100A14 is highly heterogeneous among normal human tissues, suggesting that the regulation of S100A14 expression and its function may be tissue- and context-specific. Compared to the normal counterparts, *S100A14* mRNA and protein levels have been found to be deregulated in several cancer types, indicating a functional link between S100A14 and malignancies. Accordingly, S100A14 is functionally linked with a number of key signaling molecules such as p53, p21, MMP1, MMP9, MMP13, RAGE, NF-kB, JunB, actin and HER2. Of interest, S100A14 seems to have seemingly opposite functions in malignancies arising from the gastrointestional tract (tissues rich in epithelial components) compared to cancers in the other parts of the body (tissues rich in mesenchymal components). The underlying mechanism for these observations are currently unclear and may be related to the relative abundance and differences in the type of interaction partners (effector protein) in different cancer types and tissues. In addition, several studies indicate that the expression pattern of S100A14 has a potential to be clinically useful as prognostic biomarker in several cancer types. This review attempts to provide a comprehensive summary on the expression pattern and functional roles/related molecular pathways in different cancer types. Additionally, the prognostic potential of S100A14 in the management of human malignancies will be discussed.

## INTRODUCTION

### S100 proteins

Moore identified an unfractionated mixture of S100B and S100A1 from the bovine brain and called it ‘S100’ because the mixture was soluble in 100% saturated solution of ammonium sulphate [1]. The S100 proteins are low molecular weight acidic proteins that constitute the largest subfamily of calcium (Ca^2+^)-binding protein of the EF-hand type. At least 25 closely related S100 proteins with a sequence homology of 25-65% are described. Out of 25 members, genes for 21 proteins are clustered at the chromosome locus 1q21, while the remaining 4 (*S100B*, *S100G*, *S100P* and *S100Z*) are dispersed throughout the genome [2–4]. These genes encode small (10-12 kDa) proteins with two characteristic EF-hands connected by a central hinge region [5]. The proteins are expressed in a cell- and tissue-specific manner and are involved in several biological processes related to normal development and pathological conditions including malignancies [6–11]. S100 proteins have been reported to be involved in a number of intracellular and extracellular functions. The intracellular functions include regulation of protein phosphorylation, Ca^2+^ homeostasis and activities of transcription factors; as well as the control of cell growth and differentiation, *etc* [6, 8, 12–14]. Some of the S100 members are secreted extracellularly and exert diverse biological functions in autocrine/paracrine manner via interaction with a variety of cell-surface receptors. Despite these diverse cellular functions, the S100 proteins do not possess any enzymatic activity to account for their functions. The ability of majority of the S100 proteins to interact with and to modulate the functions of effector proteins is one of the key mechanisms for their biological functions [15, 16]. Several members of the S100 family such as S100B [17–22], S100A4 [21, 23–25], S100A2 [26], S100A1 [27], S100A6 [28, 29], S100A9 [30] and S100P [31] have been shown to interact with the tumor suppressor protein p53 with variable effects on cellular functions. In addition, a number of S100 proteins can form homodimers/oligomers (for example: S100A4 [32], S100B [33] or heterodimers (for example: interactions between S100A8 and S100A9 [34], S100A4 and S100A1 [35], S100B and S100A6 [33], S100A14 and S100A16 [36], and this is considered to be important for their cellular functions. Similarly, interaction of extracellular S100 proteins with a number of cell-surface receptors like receptor for advanced glycation end products (RAGE), G protein-coupled receptors, scavenger receptors, CD166 antigen is key to several biological functions [37–40]. Interestingly, many of the S100 protein-mediated cellular functions such as cell growth, cell motility, signal transduction, transcription, apoptosis and cell survival are closely related to normal development and tumorigenesis [5, 8, 41]. In addition to these cancer related functions, observations such as occurrence of frequent structural and numerical aberrations in the chromosomal region 1q21 [42, 43] (where most of the *S100* gene members are located [2]) in human cancers; and altered mRNA and protein expression levels of several of the S100 members in different human malignancies [44–51] suggest that these proteins are closely related to human malignancies.

S100A14 (also known as Breast Cancer Membrane Protein 84) is one of the youngest members of the S100 protein family and has recently gained significant attention in cancer research. S100A14 was first identified in 2002 by analysing human lung cancer cell lines [3], and subsequently in 2003 as a membrane-associated protein in breast cancer cells [52]. S100A14 has been reported to be differentially expressed in various cancer types and has been suggested to be involved in key biological processes critical for cancerous phenotypes. However, the expression pattern and related cellular functions seem to be tissue-specific. This review will attempt to summarize the signaling pathways, expression patterns and functional roles of S100A14 in different human malignancies. In addition, the prognostic potential of S100A14 in the management of human malignancies will be discussed.

### Molecular structure of S100A14

**Figure 1:**
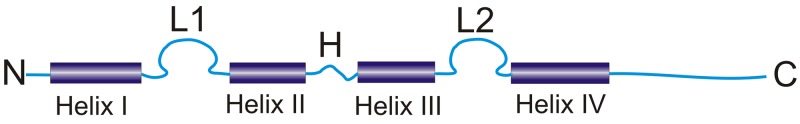
Schematic illustration of the typical structure of the S100 protein (L1 and L2: calcium-binding loops, H: hinge region, N and C: N- and C-terminals) (modified from Donato R [8])

The S100A14 gene is located on chromosome 1q21. In contrast to other S100 members which consist of 3 exons and 2 introns (except *S100A5*, consisting of 4 exons with exon 3 and 4 being the coding ones; *S100A4*, containing an additional alternatively spliced untranslated exon; and *S100A11*, with the coding sequence beginning already in the first exon), the *S100A14* contains 4 exons and 3 introns with exons 2-4 being the translated ones [3]. The *S100A14* gene encodes for a Ca^2+^ binding protein (104 amino acids) with a predicted molecular weight of 11.6 kDa [3]. Similar to other S100 protein members, S100A14 protein monomer consists of two Ca^2+^ binding motifs of the EF-hand type separated by a flexible hinge region (Figure 1). Each Ca^2+^ binding motif consists of a Ca^2+^ binding loop flanked by two α-helices. Helices I and II flank the Ca^2+^ binding loop in the N-terminal site whereas the Ca^2+^ binding loop in the C-terminal site is flanked by III and IV helices (Figure 1). Helix IV in the C-terminal site is followed by a C-terminal extension [3]. The S100A14 amino acid sequence shares significant similarity (55-68%) and identity (30-38%) with other S100 members [3]. Nevertheless, the Ca^2+^ binding loop at the N-terminal of the S100A14 protein contains 13-amino-acids loop which is in contrast to the 14-amino-acids loop characteristic of the S100 protein family. Moreover, the Ca^2+^ binding loop at the C-terminal has been reported to carry mutations thus limiting the Ca^2+^ binding ability of the S100A14 [3]. The functional implications of limited Ca^2+^ binding ability of S100A14 are currently unclear.

### Cellular signaling pathway of S100A14

**Figure 2:**
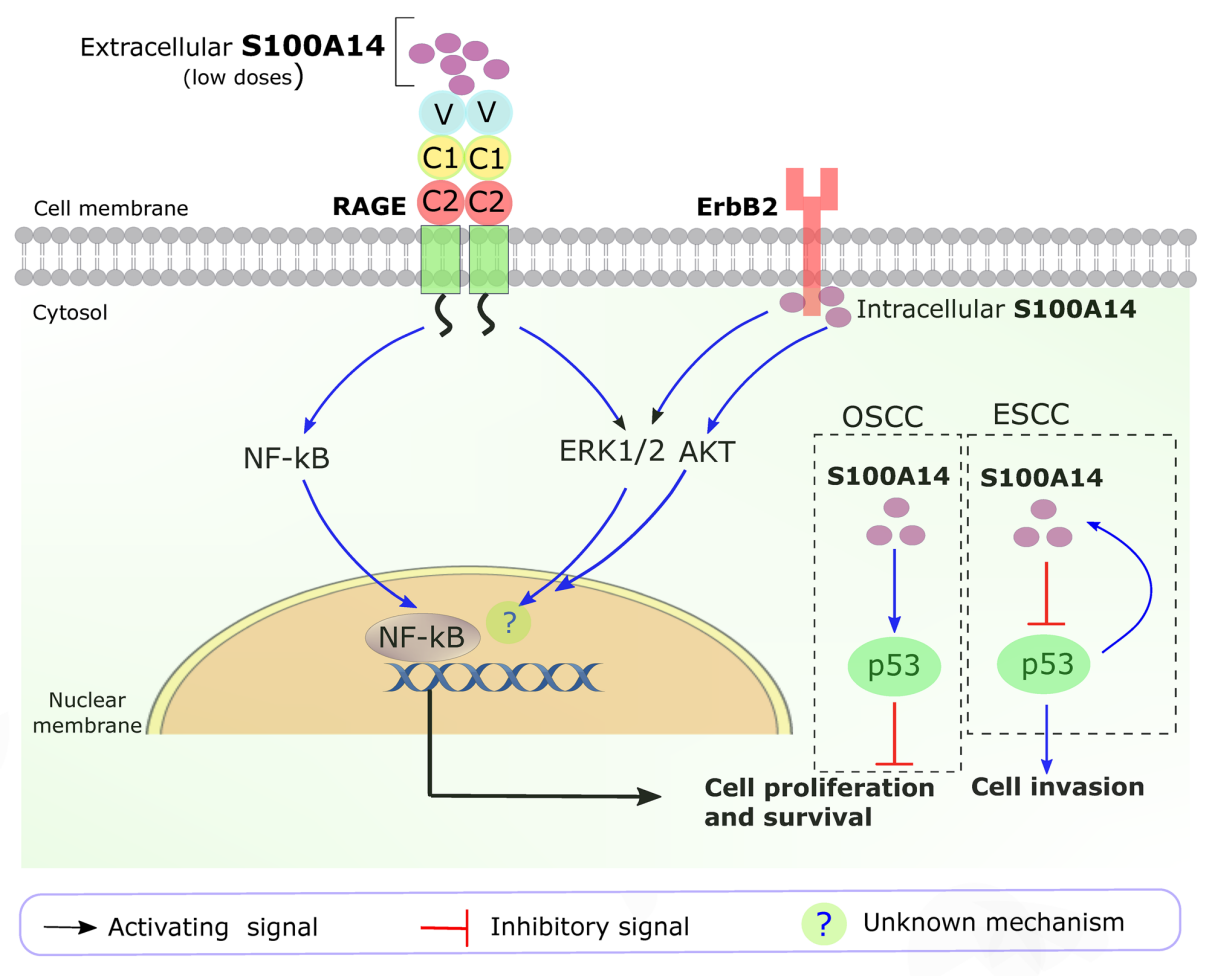
Key S100A14 signaling pathways in human cancers Extracellular S100A14 (low amount) binds with RAGE and activates ERK1/2 and NF-kB signaling pathways, leading to ESCC cell proliferation and survival. Intracellular S100A14 binds to the intracellular domain of ERbB2, leading to the activation of ERK1/2 and AKT pathways in breast cancer cells. In OSCC, S100A14 positively affects the function of p53. In ESCC, p53 positively regulates the transcription of *S100A14,* whereas S100A14 negatively regulates the expression and function of p53. OSCC, oral squamous cell carcinoma; ESCC, esophageal squamous cell carcinoma; ?, unknown mechanisms downstream of ERK1/2 and AKT pathways.

The detailed cellular signaling pathways of S100A14 are not fully understood. Considering its diverse cellular functions and seemingly opposite roles in malignancies arising from the gastrointestional tract (tissues rich in epithelial components) compared to cancers in the other parts of the body (tissues rich in mesenchymal components), it can be speculated that S100A14 is involved in a wide range of signaling pathways with a considerable variation in the outcome. Similar to many other S100 proteins, S100A14 has been shown to (i) interact with transmembrane receptors (such as RAGE, human epidermal growth factor receptor 2 (HER2 or ErbB2)) [53, 54] and other S100 protein members (for example S100A16) [36], and (ii) have a tight functional link with the tumor suppressor p53 in human cancers [10, 55] (Figure 2).

The extracellular S100A14 was shown to bind with RAGE leading to the activation of mitogen activated protein (MAP) kinase (extracellular signal-regulated kinase, ERK) and NF-κB signaling pathways, thereby promoting cell proliferation and survival in esophageal squamous cell carcinoma (ESCC) (Figure 2). However, higher concentration of extracellular S100A14 was found to induce cell apoptosis through mitochondria-mediated reactive oxygen species production, partly in a RAGE dependent manner [53]. Additionally, S100A14 was found to interact with HER2 in breast cancer cells. The interaction increased phosphorylation of HER2, thereby leading to the activation of down-stream signaling pathways (phosphorylation of AKT and ERK) contributing to breast cancer cell proliferation [54] (Figure 2).

We previously found a positive functional link between S100A14 and p53 contributing to the suppression of oral squamous cell carcinoma (OSCC) cell proliferation [10]. In contrary, a negative feedback loop between S100A14 and p53 was suggested to enhance motility and invasiveness of ESCC cells [55] (Figure 2).

## EXPRESSION PROFILE OF S100A14 IN NORMAL HUMAN TISSUES AND MALIGNANCIES

**Figure 3:**
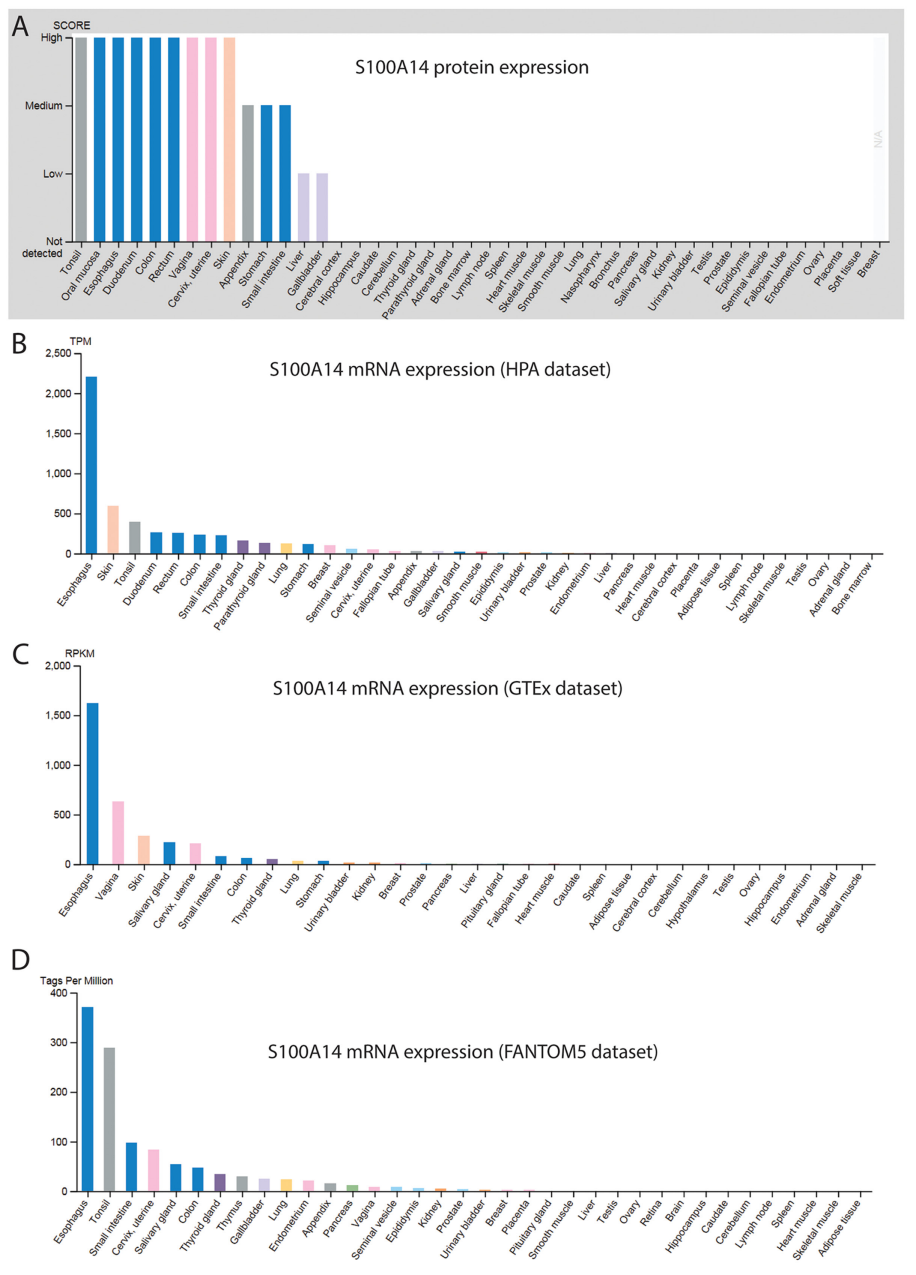
Protein **(A)** and mRNA **(B-D)** data demonstrating differential expression of S100A14 in various human normal tissues**.**

The expression profile of S100A14 in various normal human tissues and malignancies has been widely studied. Using Northern blot and Cancer profiling array, Pietas et al. showed that *S100A14* mRNA was expressed in a number of human normal tissues. Among them, tissues with epithelial-parenchymal phenotype such as colon, rectum, small intestine, stomach, thymus, thyroid, kidney and lungs were found to express higher levels of *S100A14*. In contrast, tissues with mesenchymal-stromal phenotype such as brain, white blood cells, muscles, spleen expressed negligible amounts of *S100A14* mRNA [3]. In parallel, the protein and mRNA expression data from the Human protein atlas [56, 57] (image available from https://www.proteinatlas.org/ENSG00000189334-S100A14/tissue) and other mRNA databases, (GTEx) [58] and FANTOM5 [59, 60] revealed higher expression of S100A14 in various parts of oral-digestive tract and skin (Figure 3). Interestingly, anatomical sites with higher S100A14 expression include simple or stratified epithelium as a major component of their structures. The highly heterogeneous pattern of S100A14 expression among the normal tissues suggests that regulation of S100A14 expression and its functions may be complex and tissue- and context-specific. Compared to the normal counterparts, the expression of *S100A14* mRNA/protein has been found to be deregulated in several cancer types, indicating a functional link between S100A14 and malignancies. Below we describe the expression pattern of S100A14 in some of the cancer forms where S100A14 has been well investigated (Table 1).

**Table 1:** Deregulation of S100A14 in human cancers

Cancer type	Source	mRNA/protein	Up/downregulation (cancer versus normal)	Ref.
OSCC	Cultured cells/Tissue	mRNA and protein	Down	[9, 10, 66]
Gastrointestinal cancer				
Esophageal sq. cell carcinoma	Tissue	mRNA and protein	Down	[71, 73]
Small intestinal adenocarcinoma	Tissue	Protein	Down	[76]
Colorectal carcinoma	Tissue	mRNA and protein	Down	[3, 77]
Ovarian cancer				
Epithelial ovarian cancer	Cultured cells/Tissue	mRNA and protein	Up	[3, 78]
Serious ovarian cancer	Tissue/Serum	Protein	Up	[79]
Cervical cancer	Tissue	Protein	Up	[93]
Breast cancer	Tissue	mRNA and Protein	Up	[3, 81]
Breast cancer	Tissue	Protein	Up	[54]
Lung adenocarcinoma	Tissue/Serum	Protein	Up	[83]
Lung adenocarcinoma	Tissue	Protein	Up	[84, 85]
Hepatocellular carcinoma	Tissue	Protein	Up	[82]
Uterine cancer	Tissue	mRNA	Up	[3]
Renal cancer	Tissue	mRNA	Down	[3]
Bladder cancer	Tissue	mRNA	Up	[80]

Down: downregulation; Up: upregulation.

### Oral squamous cell carcinoma (OSCC)

**Figure 4:**
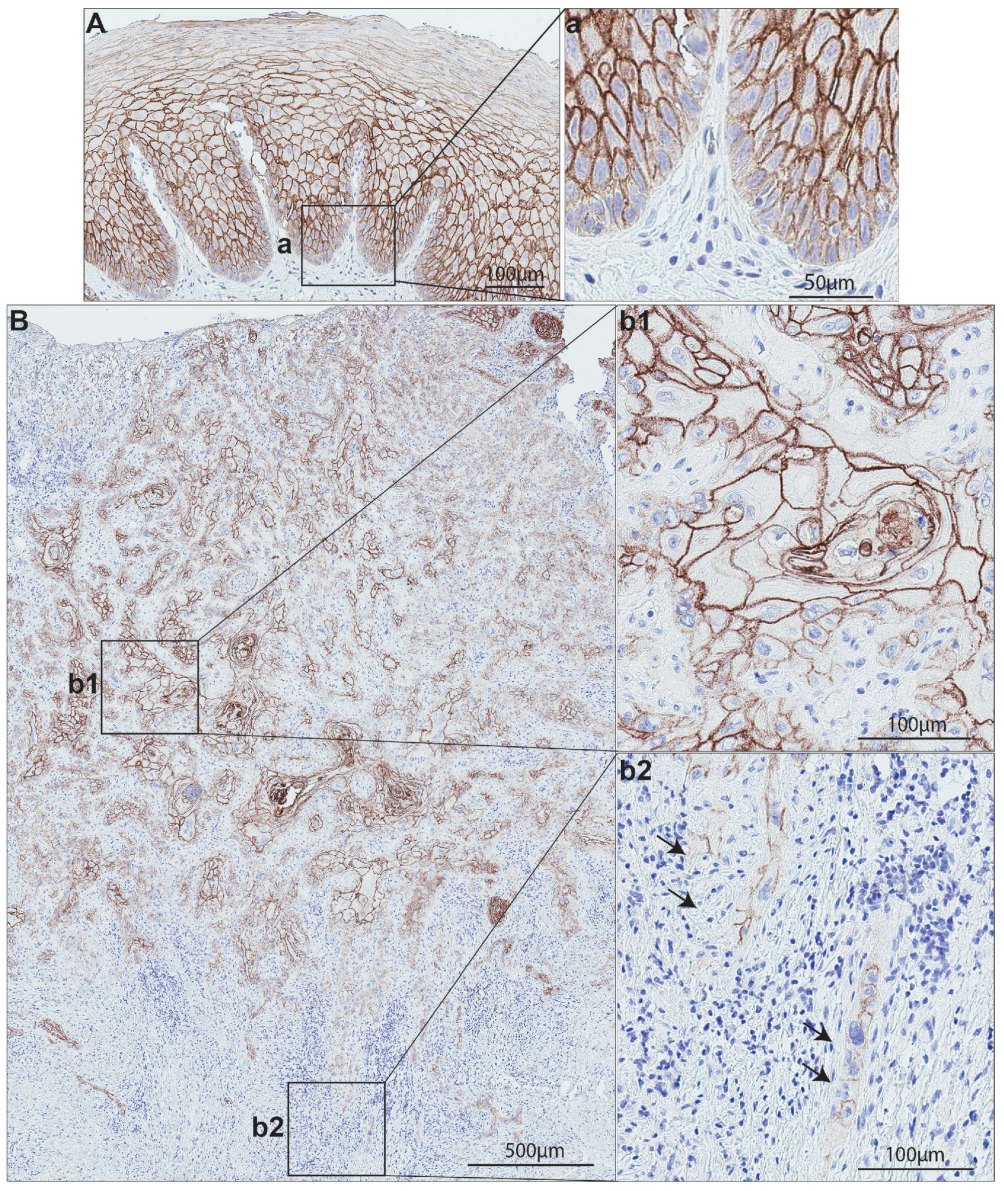
S100A14 expression on normal human oral mucosa and oral cancer specimens **(A)** Representative normal human oral mucosa specimen showed strong, predominantly membranous S100A14 expression in the epithelial compartment. **(B)** Representative oral cancer lesion showing a gradient of S100A14 expression: central area (b1) showed a strong, membranous staining in contrast to a very weak, staining in the invading front area (b2).

Oral cancer, a sub-group of head and neck cancers, refers to the malignancy arising from the mucous membranes and other tissues of oral cavity [61]. OSCC is the major type of oral cancer encompassing at least 90% of all oral malignancies [61–63]. Among the multiple types of genetic changes occurring in OSCCs, a frequent loss of chromosomal regions (including the 1q21) harboring S100 family gene members has been shown to be a common event [64, 65]. Chromosomal region harboring *S100A14* gene has been found to be frequently deleted in OSCC [64]. Accordingly, we previously reported downregulation of *S100A14* mRNA levels in OSCC specimens and OSCC-cell lines as compared to their corresponding normal controls [9, 10, 66]. Immunohistochemical analysis of normal human oral mucosa showed strong membranous expression of S100A14 protein in all suprabasal epithelial cells with low expression in the basal cells and almost no expression in the stromal compartment (Figure 4A) [9]. In contrast, OSCC specimens displayed a variable S100A14 expression pattern across different areas of the lesion. A gradual loss of S100A14 from the surface/tumor center (more differentiated areas) to the invading front/island (poorly differentiated areas) was observed in OSCC samples (Figure 4B), following the same pattern for decreased gradient of expression from surface to the basal cell layer as observed in the normal oral mucosa. Interestingly, a change in the sub-cellular localization, from predominantly membranous staining seen in the central areas of the OSCC to mixed membranous and cytoplasmic staining was found at the invading tumor islands (Figure 4B) [9]. In parallel to these findings, a gradual loss of *S100A14* mRNA and protein was found as well in an *in vitro* OSCC-progression model consisting of normal, dysplastic (premalignant) and cancerous oral keratinocyte cell lines [9, 10]. Taken together, downregulation of S100A14 seems to be a common molecular alteration in OSCC. One of the mechanisms responsible for this finding could be the loss of chromosomal region 1q21 harboring *S100A14* gene. However, the heterogeneity of the protein expression in the same lesion and the common pattern for decreased gradient of protein expression observed in both normal and neoplastic mucosa might indicate a more complex, posttranslational regulation of S100A14 expression, governed by hierarchical cellular processes of differentiation. On the other hand, occurrence of hypermethylated CpG islands in the promotor region, a common occurrence for some of the S100 members [67–70], seems to be a less likely mechanism for *S100A14* downregulation in OSCC as no CpG islands have been reported in 5’ upstream region and introns of *S100A14* [3]. In support of this line of thinking are also findings from *in vitro* models of another epithelial malignancy which revealed that treatment with 5-aza-2’deoxycytidine could not induce re-expression of *S100A14* mRNA in eight lung cancer cell lines [3].

### Esophageal squamous cell carcinoma (ESCC)

The expression levels of both *S100A14* mRNA and protein were reported to be significantly downregulated in ESCC compared to the normal adjacent epithelial tissue [71–73]. Using S100A14 immunohistochemistry, Chen et al. found a predominantly membranous staining in the keratinocytes in normal esophageal epithelium. However, a mixed staining pattern (plasma membranous and cytoplasmic) was reported in ESCC specimens. Interestingly, similar to our previous observation in OSCC, a stronger S100A14 expression was reported in more differentiated (keratinized) areas as compared to the less differentiated (invading areas) of ESCC [71, 73], suggesting again a functional link between S100A14 and keratinocyte differentiation.

### Other gastrointestinal carcinomas

#### Gastric cancer

The expression of S100A14 in gastric carcinoma appears to be variable as compared to the normal control. Zhu et al. investigated the expression of S100A14 in a large cohort of gastric cancer specimens (n=485) with pair-wised matched control gastric mucosa (n=289). Nevertheless, the authors did not find any significant difference in S100A14 expression between gastric cancers and matched controls [74]. However, Zhang et al. reported a differential expression of S100A14 in gastric carcinoma as compared to controls (S100A14 was upregulated in some gastric carcinomas, while downregulated in others) [75].

#### Small intestine adenocarcinoma (SIAC)

Using immunohistochemistry, Kim et al. reported a downregulated expression of S100A14 in SIACs as compared to normal intestinal epithelium. S100A14 was mostly expressed in cell membrane of normal small intestinal mucosal epithelial cells. The expression was pronounced on the surface epithelial cells and reduced in the crypts, with no expression in Brunner’s glands. Out of 175 SIACs, loss of S100A14 was reported in 73% of the cases. The loss of expression was common mainly in the ulceroinfiltrative and polyploid tumors compared to flat tumors. In addition, the loss was more frequent in distal (ileum or jejunum) tumors of the intestine than proximal (duodenum) ones [76].

#### Colorectal cancer (CRC)

Downregulation of S100A14 is reported in majority of the CRC samples, however, a significant proportion of the CRC samples has also been reported to express higher levels of S100A14. Using RT-PCR, Wang et al. reported both downregulation (52.5%) and upregulation (47.5%) of *S100A14* mRNA in primary colorectal cancer (n=40) as compared to their matched normal mucosa. In line with the mRNA data from RT-PCR, S100A14 immunohistochemistry showed downregulation or loss of S100A14 expression in majority (56.5%) of colorectal cancer. Membranous S100A14 staining was prominent in the well-differentiated areas in the tumor. The author reported a substantial expression of S100A14 in different human colorectal cancer cell lines (SW840, SW620, LoVo and HT29) [77].

### Ovarian cancers

Overall, S100A14 has been reported to be overexpressed in ovarian cancers as compared to the normal control tissues. Using immunohistochemistry in formalin-fixed paraffin embedded specimens of ovarian cancers (n=71), borderline ovarian tumors (n=10), mucinous cystadenomas (n=10) and normal ovary tissues (n=13), Cho et al. reported a gradual over-expression of S100A14 from normal tissues to benign, borderline and ovarian carcinomas [78]. In parallel, Qian et al. reported an upregulation of S100A14 in serous ovarian carcinomas compared to normal controls [79]. Both authors found predominant cytoplasmic S100A14 staining in carcinoma cells. In addition, over-expression of *S100A14* mRNA and protein in ovarian cancer cell lines as compared to human ovarian surface epithelial cells has been reported [78].

### Bladder cancer

Yao et al. reported an upregulation of *S100A14* mRNA in 4-hydroxybutyl(butyl)nitrosamine-induced mouse and human bladder cancers compared to their corresponding controls [80].

### Breast cancers

S100A14 has been reported to be upregulated in breast cancers. Tanaka et al. performed immunohistochemical analysis of S100A14 in archival specimens of primary tumors of 167 breast cancer patients and found that 53% of cases expressed strong, predominantly membranous S100A14, while the control normal tissue showed no or faint staining [81]. Similarly, Xu et al. found strong membranous S100A14 staining in breast cancer specimens (n=74) compared to their matched control specimens. In addition to the tissue specimens, the assessment of cell lines suggests a predominant plasma membrane (inner side) localization of S100A14 [54]. Interestingly, Tanaka et al. showed that S100A14 expression was polarized to the lateral and the intercellular junctions but not to the basal or the apical region, indicating that S100A14 potentially binds to membrane-associated proteins [81].

### Hepatocellular carcinoma

Similar to breast, ovarian and bladder cancers, overexpression of S100A14 has been reported in hepatocellular carcinoma. Using immunohistochemistry on specimens of hepatocellular carcinoma (n=120) and their corresponding paratumor normal liver tissue (n=14), Zaho and co-workers found a higher S100A14 expression in carcinoma compared to the controls. Interestingly, a nuclear or cytoplasmic localization of S100A14 was reported in carcinoma cells [82].

### Lung adenocarcinoma

S100A14 has been reported to be over-expressed in lung adenocarcinomas as compared to the normal control tissues. A predominantly membranous staining was commonly found in the carcinoma cells [83–85].

## FUNCTIONAL ROLE OF S100A14 IN HUMAN CANCERS

S100A14 has been suggested to be involved in a number of biological pathways (cell proliferation and apoptosis, cell migration and invasion, differentiation, *etc.*) related to carcinogenesis. However, in parallel to the differential expression pattern and sub-cellular localization in different cancer types, the S100A14-mediated functions seem to be specific with respect to cancer types. In some tumor types, S100A14 seems to function as a tumor suppressor protein while in others it seems to promote tumorigenesis. Below we will summarize the functional roles and related molecular pathways of S100A14 in major cancer types.

### Oral cancer

**Figure 5:**
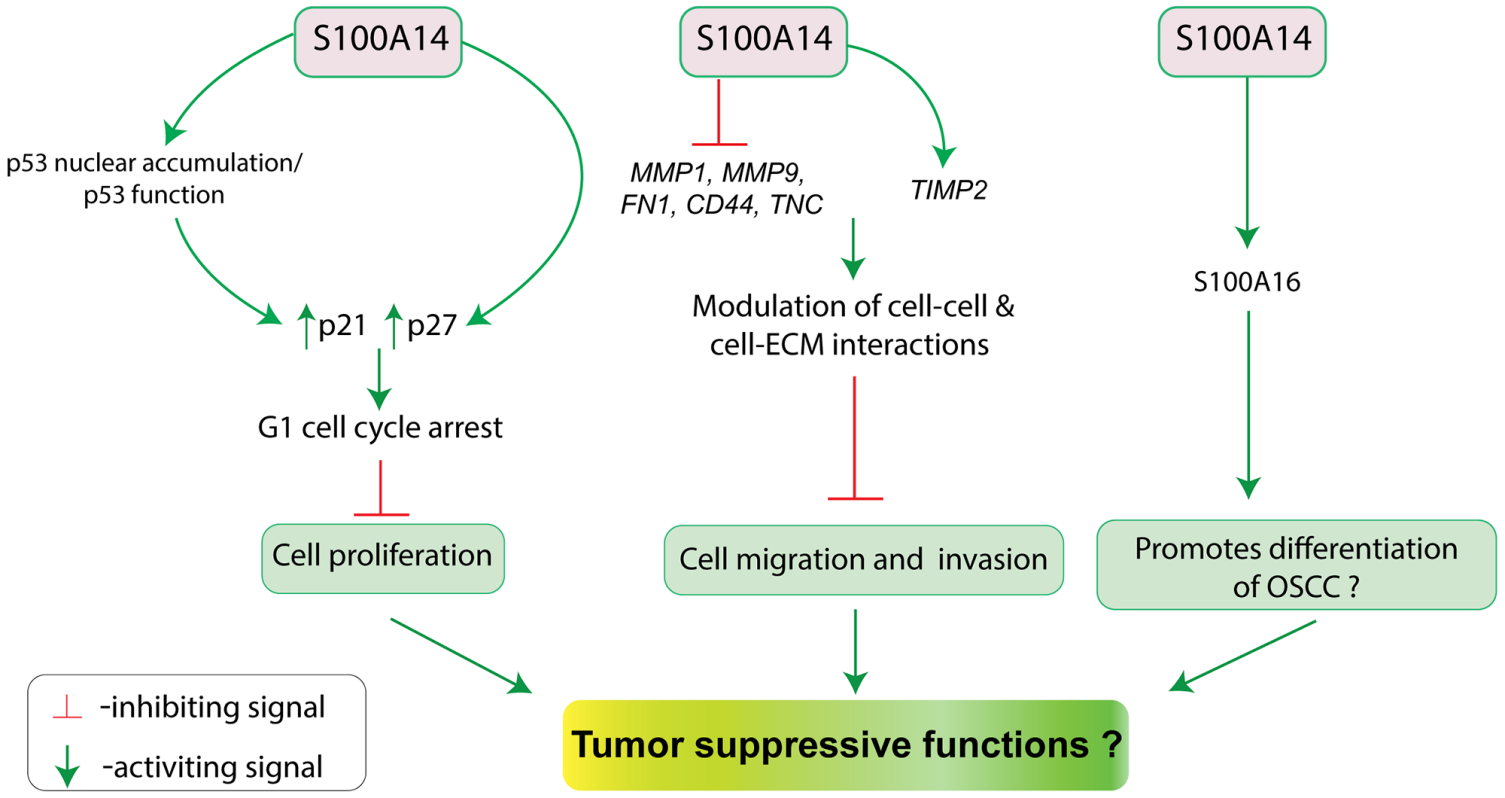
Schematic illustration for the putative tumor suppressive functions (inhibition of cell proliferation, migration and invasion; promotion of differentiation?) of S100A14 in oral cancer

Based on the progressive downregulation of *S100A14* mRNA and protein from normal oral mucosa to OSCC, we previously hypothesized that S100A14 might be related to tumor suppressive functions in OSCC (Figure 5). Subsequent studies showed that S100A14 might indeed function as a tumor suppressor by inhibiting OSCC cell proliferation and/or invasion [9, 10].

Retroviral-mediated over-expression of S100A14 in OSCC cell lines (CaLH3 and OSCC1) harboring wild type p53 led to reduced cell proliferation and G1-cell cycle arrest with concomitant upregulation of the cell cycle inhibitor p21 and p27 proteins. As the tumor suppressor protein p53 is one of the key regulators of p21 expression [86], a possible link between p53 and S100A14-mediated upregulation of p21 was examined by using shRNA-mediated knock-down of p53. Partial suppression of p21 expression with p53 knock-down suggested that S100A14 over-expression mediated upregulation of p21 and associated G1-phase cell cycle arrest might, at least, be partly dependent on the activity of the WT p53 in CaLH3 and OSCC cells. Additionally, S100A14 was found to enhance nuclear accumulation of p53 in OSCC cells. Taken together, S100A14 was found to inhibit OSCC cell proliferation by inducing G1-cell cycle arrest, partly, through p21-p53 axis [10].

Significant downregulation of S100A14 particularly at the invasive tumor islands of OSCC led us to examine the role of S100A14 in the regulation of invasive phenotype of OSCC cells. Experimentally, retroviral-mediated over-expression of S100A14 resulted in significant decrease of the migratory and invasive phenotype whereas siRNA mediated knock-down led to opposite effects in OSCC cells. The reduction in the invasive potential of OSCC cells was associated with the concomitant downregulation of matrix metalloproteinase (MMP) like *MMP1* and *MMP9* mRNAs, and suppression of *MMP9* gelatinolytic activity [9]. These observations suggest that S100A14 might contribute to less aggressive OSCC behavior by suppressing the invasion potential of OSCC cells.

Although not yet experimentally proven, the finding that S100A14 expression was significantly lost at the invading front/island (poorly differentiated areas) of OSCC as compared to the tumor surface/tumor center (more differentiated areas), suggests a functional link between S100A14 and cellular differentiation. Indeed, there are several observations which indicate a role for S100A14 in cellular differentiation. Firstly, genes encoding several S100 proteins including S100A14 are clustered in the epidermal differentiation complex on chromosome 1q21 and many of the S100 members have been reported to be involved in cellular differentiation and differentiation-related pathologies [87, 88]. Secondly, S100A14 has been shown to promote differentiation of ESCC and gastric cancer cells [73, 74]. Thirdly, S100A14 was found to interact with and regulate the expression of S100A16 (another member of S100 protein family), which suppresses the OSCC progression by promoting OSCC cell differentiation [89]. Hence, future studies looking for the expression of differentiation markers in OSCC cells with modulation of S100A14 expression might reveal a link between S100A14 and OSCC differentiation.

### ESCC

Sequencing analysis of *S100A14* gene and untranslated regions has shown that genetic variants can occur in *S100A14* gene and that might influence S100A14-mediated cellular functions and susceptibility for ESCC development. Further, in-depth functional studies have linked S100A14 to the regulation of differentiation, proliferation, apoptosis and invasive potential of ESCC cells.

Chen et al. reported that a S100A14 gene (461G>A) variant in 5’ untranslated region contributed to reduced expression of S100A14 in ESCC, probably due to reduced transcriptional activity mediated by p53. Additionally, the genetic variant 461G>A was found to be associated with susceptibility to ESCC in a Chinese population with smoking habit [72]. In contrary, another study by Zhao et al. in Chinese population showed that the same genetic variant (rs11548103G>A) can have a protective effect in ESCC carcinogenesis [90]. These results warrant further studies including a sufficiently large number of samples and with detailed clinicopathological information to clarify the association between S100A14 genetic variant and ESCC carcinogenesis.

In parallel to the observation that more differentiated ESCC expressed higher expression of S10014 in clinical specimens, Chen et al. reported increased expression of S100A14 in 12-O-tetra-decanoylphorbol-13-acetate and Ca^2+^-induced more differentiated ESCC cells [73]. Further, S100A14 expression was found to regulate the expression of terminal differentiation markers and induce G1-phase cell cycle arrest. Additionally, the authors suggested that JunB could transcriptionally regulate S100A14 expression and this regulation might be important in S100A14-mediated ESCC differentiation [73]. On the other hand, the effect of exogenous treatment of S100A14 seems to display different cellular functions based on the amount of S100A14. Low amount of S100A14 activated ERK1/2 and NF-kB signaling thereby stimulating ESCC cell proliferation or survival via RAGE signaling. However, higher amount resulted in increased production of reactive oxygen species in a RAGE-dependent manner [53].

In addition to these cellular functions, Chen et al. have linked S100A14 with ESCC invasion. The authors showed that overexpression of S100A14 promoted motility and invasiveness of ESCC, at least partly through upregulating the expression and activity of MMP2 in a p53-dependent manner. Moreover, based on the observation that expression of S100A14 is dependent on the p53 regulation and that S100A14 decreases the expression of p53, the authors suggested a negative feedback loop between S100A14 and p53 [55]. In contrary, our previous work suggested a positive mutual functional regulation between S100A14 and p53 in OSCC cells [10]. A positive mutual regulation has also been suggested for S100A2 and p53 [26, 91]. Nevertheless, the discrepancy in the nature of mutual regulation between S100 protein members and p53 between oral and esophageal epithelial cells needs further investigation.

### Other gastrointestinal carcinomas

Expression analyses have found a correlation between loss/downregulation of S100A14 in gastrointestinal cancers and disease aggressiveness as well as poor patient outcomes [74, 76, 77]. In parallel with these observations, S100A14 has been reported to regulate differentiation and migratory/invasive potential of gastric cancer cells [74, 75]. Zhu et al. found that S100A14 was positively correlated with more differentiated phenotype of gastric cancer cells with concomitant regulation of E-cadherin and PGII expression. Additionally, expression of S100A14 negatively regulated the migratory/invasive potential of gastric carcinoma cells *in vitro* and *in vivo*. The authors, using a series experiments, further suggested that the reduced invasive potential was related to the concomitant activation and stabilization of FAK, and downregulation of MMPs (MMP2, MMP9 and MMP11), probably mediated by the inhibition of store-operated Ca^2+^ influx in the gastric carcinoma cells [74].

### Ovarian cancer

Ovarian cancer specimens with positivity or upregulation of S100A14 have been correlated with advanced stage, poor tumor grade, tumor recurrence, resistance to platinum-based therapy and shorter survival probabilities [78, 79, 92]. By using lentiviral-mediated expression and knock-down strategies, Cho and coauthors have investigated the role of S100A14 in the malignant phenotype of epithelial ovarian cancer cells. The authors reported increased cell proliferation, colony and sphere formation abilities, and migration and invasion of epithelial ovarian cancer cells upon S100A14 over-expression. The opposite results have been shown with the knock-down of S100A14. In line with these *in vitro* results, S100A14 was found to increase the growth of subcutaneous tumor xenograft in mice. Investigation of the underlying signaling pathway revealed that S100A14 might contribute to epithelial ovarian cancer progression via PI3K/Akt pathway [78].

### Cervical cancer

Wang et al. found a positive correlation between higher expression of S100A14 with poor prognosis in cervical cancer specimens. To investigate the biological roles of S100A14 in cervical cancer progression, the authors used lentiviral-mediated modulation of the endogenous S100A14 in cervical cancer cells. The modulation of S100A14 correlated positively with increased proliferation, migration and invasion of cervical cancer cells *in vitro*. The increased cell proliferation was associated with reduced proportion of cells in G1- and increased proportion in G2/M- phase of cell cycle. Further, S100A14 was shown to enhance epithelial mesenchymal transition of cervical cancer cells as evidenced by the upregulation of N-cadherin and Vimentin, and downregulation of E-cadherin [93].

### Breast cancer

In breast cancer tissues and cell lines, S100A14 has been found to co-localize with HER2 [54] and actin proteins [81]. Immunoprecipitation studies have shown that S100A14 indeed interacts with HER2 [54] and actin [81]. The interaction between S100A14 and HER2 seems to be Ca^2+^-dependent as treatment with 1mM Egtazic acid was found to inhibit the interaction [54]. Nevertheless, interaction with actin seems to be independent of Ca^2+^ as increasing the concentration of Ca^2+^ did not increase immunoprecipitation [81]. The latter is in line with our previous study where increasing Ca^2+^ concentration did not enhance interaction. One of the possible explanation for this observation could be the fact that S100A14 is a poor Ca^2+^ binder with semi-open conformation even in apo-state and this conformation might not necessarily change even after binding Ca^2+^ ions [94].

These interactions have been suggested to have a functional significance in breast cancer cells. For example, Tanaka et al. suggested that S100A14 may promote cancer invasion by binding to the actin, thereby activating cell movement [81]. Similarly, Xu et al. examined the functional significance of S100A14 and HER2 by silencing S100A14, and found a subsequent reduction in HER2 phosphorylation and downstream signaling with concomitant reduction in the HER2-stimulated breast cancer cell proliferation [54]. Furthermore, several other studies have supported the role of S100A14 in breast cancer progression [95, 96]. These observations indicate that one of the ways that S100A14 executes its diverse functions in breast cancer cells could be through molecular interaction with other cellular proteins involved in key biological pathways.

### Hepatocellular carcinoma

Zhao et al. investigated the functional roles of S100A14 in hepatocellular carcinoma cells by using S100A14 over-expression or knock-down strategies [82]. A decrease in cell proliferation and invasion was found with knock-down of S100A14 in hepatocellular carcinoma cells *in vitro* [82, 97]. Opposite effects were reported with S100A14 over-expression. In parallel with these *in vitro* data, smaller subcutaneous tumor xenografts and significantly reduced number of lung metastases were found in severe combined immunodeficient mice with knock-down of S100A14. Although no molecular mechanisms have been suggested, these results indicate that S100A14 might promote hepatocellular carcinoma [82].

### Lung adenocarcinoma

Biological functions of S100A14 in lung adenocarcinoma have not been well investigated as compared to oral cancer or ESCC. Ken et al. used siRNA technique to downregulate the expression of S100A14 in LC-2/ad cells and showed that downregulation of S100A14 was correlated with less migratory (scratch assay) and less invasive (Matrigel invasion assay) phenotype [84]. This observation is in line with association of high S100A14 expression in lung adenocarcinoma specimens with lymph node metastasis, intratumoral vascular invasion, intratumoral lymphatic invasion, pleural invasion [83, 84].

## S100A14 AS A POTENTIAL PROGNOSTIC MARKER AND THERAPEUTIC TARGET IN HUMAN CANCERS

Several studies have used immunohistochemistry to examine the prognostic potential of S100A14 in different human cancers. It is interesting to note that higher or positive S100A14 expression in the carcinomas of gastrointestinal tract was found to be correlated with better patient survival. In contrary, the higher expression of S100A14 in carcinomas from other organs such as ovary, breast, cervix, lung and liver seem to predict poorer survival. S100A14 expression pattern and correlation with clinicopathological and prognostic parameters in different cancer types is summarized in Table 2.

**Table 2:** Association of S100A14 protein expression with clinicopathological and prognostic variables in human cancers

Cancer type	*n*	Association with clinical/prognostic variables	Ref.
Gastrointestinal cancer			
Esophageal sq. cell carcinoma	110	low S100A14 correlated with poor differentiation and higher clinical stage	[73]
Gastric cancer	485	high S100A14 positively correlated with Lauren classification and differentiation and better OS^a^, but negatively correlated with tumor depth, lymph node status and distant metastasis	[74]
Gastric cancer	79	high S100A14 positively correlated with differentiation and survival	[75]
Small intestinal adenocarcinoma	175	-ve S100A14 correlated with lymph node metastasis and advanced disease stage	[76]
Colorectal carcinoma	115	-ve S100A14 correlated with poor differentiation, distance metastasis and shorter OS^b^	[77]
Ovarian cancer			
Epithelial ovarian cancer	71^*^	+ve S100A14 correlated with advanced stage and poor tumor grade, shorter DFS and OS^c^	[78]
Serious ovarian cancer	127	high S100A14 correlated with tumor recurrence and lymph node metastasis, shorter OS^c^	[92]
Serious ovarian cancer	125	high S100A14 correlated with resistance to platinum based therapy	[79]
Cervical cancer	98	high S100A14 correlated with FIGO stage and Lymph node metastasis	[93]
Breast cancer	167	high S100A14 correlated with ER-negative, HER-2 positive and shorter OS^c^	[81]
Breast cancer	148	high S100A14 correlated with poorer DFS and OS^c^	[99]
Lung adenocarcinoma	208	high S100A14 correlated with poor differentiation, metastasis, stage^d^	[83]
Lung adenocarcinoma	166	+ve S100A14 correlated with poor differentiation, node metastasis, stage, tumor size, vascular and lymphatic invasion, shorter survival^e^	[84]
Lung adenocarcinoma	62	+ve S100A14 correlated with well/moderate differentiation	[85]
Hepatocellular carcinoma	120	+ S100A14 correlated with multiple tumor nodes, high Edmondson-Steiner grade, vascular invasion, shorter DFS and OS^c^	[82]

*n*: sample size; Ref.: references; OS: overall survival; DFS: disease free survival; +ve: positive; -ve: negative; MVA: multivariate analysis

^a^ No univariate and multivariate cox analysis was presented.

^b^ Only multivariate cox analysis results presented.

^r^ OS: significant correlation both with the univariate and multivariate cox analysis.

^d^ The authors did not find any significant association of S100A14 expression with survival in their own IHC data. However, they have reported, using Kaplan Meier plotter online tool, that high S100A14 expression significantly correlated with reduced overall and post progression survival.

^e^ Significant correlation only with univariate and not with multivariate cox analysis.

^*^ Only 65 cases were used for survival analysis.

Results from the *in vitro* and *in vivo* functional studies in several cancer types indicate that S100A14 might be useful as a therapeutic target for the treatment of malignancies. Malignancies where S100A14 functions as a tumor promotor [95, 97], strategies aimed to downregulate the S100A14 expression might be employed. On the other hand, overexpression strategies might be useful in malignancies [9, 10, 73, 74] where S100A14 functions as a tumor suppressor. Indeed, the differentiation promoting properties of S100A14 in ESCC and CRC are of immense interest for differentiation therapy. The differentiation therapy, forcing the proliferative tumor cells towards more differentiated and mature phenotype, has been realized long before and some of the drugs, such as retinoic acid and histone deacetylase inhibitors are currently in clinical use in the treatment of human malignancies [98]. Further molecular characterization of S100A14 mediated tumor suppressive functions might contribute to the better understanding of cancer biology and provide opportunity for S100A14 based therapeutics.

## CONCLUSIONS

S100A14 is differentially expressed in various normal human tissues and its deregulated expression seems to be a common event in human carcinogenesis. Functionally, S100A14 has been linked to the regulation of a number of cellular functions related to carcinogenesis, such as cell proliferation and apoptosis, tumor cell migration and invasion, and keratinocyte differentiation. It is, however, interesting to note that S100A14 seems to have a tumor suppressive functions in cancers arising from the oro-gastrointestional tract (tissues rich in epithelial components) compared to cancers in the other parts of the body (tissues rich in mesenchymal components) where it is related to the tumor promotive functions. Although the underlying mechanism for this observation are currently unclear, it can be speculated that S100A14-mediated functions are dependent on the relative abundance and the type of the interaction partners (effector protein) thereby activating different downstream signaling pathways leading to the opposite functions.

Expression pattern of the S100A14 in different cancer types has a potential to be clinically useful as prognostic biomarker in the management of human cancers. Nevertheless, studies involving a larger and well-characterized patient cohorts and with subsequent validations are clearly warranted.

## Abbreviations

BCMP84: Breast Cancer Membrane Protein 84
Ca^2+^: Calcium
CRC: Colorectal cancer
ERK: Extracellular signal-Regulated Kinase
ESCC: Esophageal Squamous Cell Carcinoma
HER2: Human Epidermal Growth Factor Receptor 2
MAP: Mitogen Activated Protein
MMP: Matrix Metalloproteinase
OSCC: Oral Squamous Cell Carcinoma
RAGE: Receptor for Advanced Glycation End Products
SIAC: Small Intestine Adenocarcinoma

## References

[1] Moore BW (1965). A soluble protein characteristic of the nervous system. Biochem Biophys Res Commun.

[2] Schäfer BW, Wicki R, Engelkamp D, Mattei MG, Heizmann CW (1995). Isolation of a YAC clone covering a cluster of nine S100 genes on human chromosome 1q21: rationale for a new nomenclature of the S100 calcium-binding protein family. Genomics.

[3] Pietas A, Schlüns K, Marenholz I, Schäfer BW, Heizmann CW, Petersen I (2002). Molecular cloning and characterization of the human S100A14 gene encoding a novel member of the S100 family. Genomics.

[4] Santamaria-Kisiel L, Rintala-Dempsey AC, Shaw GS (2006). Calcium-dependent and -independent interactions of the S100 protein family. Biochem J.

[5] Marenholz I, Heizmann CW, Fritz G (2004). S100 proteins in mouse and man: from evolution to function and pathology (including an update of the nomenclature). Biochem Biophys Res Commun.

[6] Schäfer BW, Heizmann CW (1996). The S100 family of EF-hand calcium-binding proteins: functions and pathology. Trends Biochem Sci.

[7] Barraclough R (1998). Calcium-binding protein S100A4 in health and disease. Biochim Biophys Acta.

[8] Donato R (2001). S100: a multigenic family of calcium-modulated proteins of the EF-hand type with intracellular and extracellular functional roles. Int J Biochem Cell Biol.

[9] Sapkota D, Bruland O, Costea DE, Haugen H, Vasstrand EN, Ibrahim SO (2011). S100A14 regulates the invasive potential of oral squamous cell carcinoma derived cell-lines *in vitro* by modulating expression of matrix metalloproteinases, MMP1 and MMP9. Eur J Cancer.

[10] Sapkota D, Costea DE, Blø M, Bruland O, Lorens JB, Vasstrand EN, Ibrahim SO (2012). S100A14 inhibits proliferation of oral carcinoma derived cells through G1-arrest. Oral Oncol.

[11] Heizmann CW, Fritz G, Schäfer BW (2002). S100 proteins: structure, functions and pathology. Front Biosci.

[12] Donato R (1999). Functional roles of S100 proteins, calcium-binding proteins of the EF-hand type. Biochim Biophys Acta.

[13] Zimmer DB, Cornwall EH, Landar A, Song W (1995). The S100 protein family: history, function, and expression. Brain Res Bull.

[14] Baudier J, Glasser N, Gerard D (1986). Ions binding to S100 proteins. I. Calcium- and zinc-binding properties of bovine brain S100 alpha alpha, S100a (alpha beta), and S100b (beta beta) protein: Zn2+ regulates Ca2+ binding on S100b protein. J Biol Chem.

[15] Kligman D, Hilt DC (1988). The S100 protein family. Trends Biochem Sci.

[16] Zimmer DB, Wright Sadosky P, Weber DJ (2003). Molecular mechanisms of S100-target protein interactions. Microsc Res Tech.

[17] Baudier J, Delphin C, Grunwald D, Khochbin S, Lawrence JJ (1992). Characterization of the tumor suppressor protein p53 as a protein kinase C substrate and a S100b-binding protein. Proc Natl Acad Sci U S A.

[18] Rustandi RR, Baldisseri DM, Weber DJ (2000). Structure of the negative regulatory domain of p53 bound to S100B(betabeta). Nat Struct Biol.

[19] Rustandi RR, Drohat AC, Baldisseri DM, Wilder PT, Weber DJ (1998). The Ca(2+)-dependent interaction of S100B(beta beta) with a peptide derived from p53. Biochemistry.

[20] Delphin C, Ronjat M, Deloulme JC, Garin G, Debussche L, Higashimoto Y, Sakaguchi K, Baudier J (1999). Calcium-dependent interaction of S100B with the C-terminal domain of the tumor suppressor p53. J Biol Chem.

[21] Fernandez-Fernandez MR, Veprintsev DB, Fersht AR (2005). Proteins of the S100 family regulate the oligomerization of p53 tumor suppressor. Proc Natl Acad Sci U S A.

[22] Wilder PT, Lin J, Bair CL, Charpentier TH, Yang D, Liriano M, Varney KM, Lee A, Oppenheim AB, Adhya S, Carrier F, Weber DJ (2006). Recognition of the tumor suppressor protein p53 and other protein targets by the calcium-binding protein S100B. Biochim Biophys Acta.

[23] Chen H, Fernig DG, Rudland PS, Sparks A, Wilkinson MC, Barraclough R (2001). Binding to intracellular targets of the metastasis-inducing protein, S100A4 (p9Ka). Biochem Biophys Res Commun.

[24] Grigorian M, Andresen S, Tulchinsky E, Kriajevska M, Carlberg C, Kruse C, Cohn M, Ambartsumian N, Christensen A, Selivanova G, Lukanidin E (2001). Tumor suppressor p53 protein is a new target for the metastasis-associated Mts1/S100A4 protein: functional consequences of their interaction. J Biol Chem.

[25] Orre LM, Panizza E, Kaminskyy VO, Vernet E, Gräslund T, Zhivotovsky B, Lehtiö J (2013). S100A4 interacts with p53 in the nucleus and promotes p53 degradation. Oncogene.

[26] Mueller A, Schäfer BW, Ferrari S, Weibel M, Makek M, Höchli M, Heizmann CW (2005). The calcium-binding protein S100A2 interacts with p53 and modulates its transcriptional activity. J Biol Chem.

[27] Garbuglia M, Verzini M, Rustandi RR, Osterloh D, Weber DJ, Gerke V, Donato R (1999). Role of the C-terminal extension in the interaction of S100A1 with GFAP, tubulin, the S100A1- and S100B-inhibitory peptide, TRTK-12, and a peptide derived from p53, and the S100A1 inhibitory effect on GFAP polymerization. Biochem Biophys Res Commun.

[28] Graczyk A, Słomnicki LP, Leśniak W (2013). S100A6 competes with the TAZ2 domain of p300 for binding to p53 and attenuates p53 acetylation. J Mol Biol.

[29] Słomnicki LP, Nawrot B, Leśniak W (2009). S100A6 binds p53 and affects its activity. Int J Biochem Cell Biol.

[30] Li C, Chen H, Ding F, Zhang Y, Luo A, Wang M, Liu Z (2009). A novel p53 target gene, S100A9, induces p53-dependent cellular apoptosis and mediates the p53 apoptosis pathway. Biochem J.

[31] Gibadulinova A, Pastorek M, Filipcik P, Radvak P, Csaderova L, Vojtesek B, Pastorekova S (2016). Cancer-associated S100P protein binds and inactivates p53, permits therapy-induced senescence and supports chemoresistance. Oncotarget.

[32] Novitskaya V, Grigorian M, Kriajevska M, Tarabykina S, Bronstein I, Berezin V, Bock E, Lukanidin E (2000). Oligomeric forms of the metastasis-related Mts1 (S100A4) protein stimulate neuronal differentiation in cultures of rat hippocampal neurons. J Biol Chem.

[33] Yang Q, O’Hanlon D, Heizmann CW, Marks A (1999). Demonstration of heterodimer formation between S100B and S100A6 in the yeast two-hybrid system and human melanoma. Exp Cell Res.

[34] Teigelkamp S, Bhardwaj RS, Roth J, Meinardus-Hager G, Karas M, Sorg C (1991). Calcium-dependent complex assembly of the myeloic differentiation proteins MRP-8 and MRP-14. J Biol Chem.

[35] Tarabykina S, Kriajevska M, Scott DJ, Hill TJ, Lafitte D, Derrick PJ, Dodson GG, Lukanidin E, Bronstein I (2000). Heterocomplex formation between metastasis-related protein S100A4 (Mts1) and S100A1 as revealed by the yeast two-hybrid system. FEBS Lett.

[36] Sapkota D, Costea DE, Ibrahim SO, Johannessen AC, Bruland O (2013). S100A14 interacts with S100A16 and regulates Its Expression in Human Cancer Cells. PLoS One.

[37] von Bauer R, Oikonomou D, Sulaj A, Mohammed S, Hotz-Wagenblatt A, Gröne HJ, Arnold B, Falk C, Luethje D, Erhardt A, Stern DM, Bierhaus A, Nawroth PP (2013). CD166/ALCAM mediates proinflammatory effects of S100B in delayed type hypersensitivity. J Immunol.

[38] Dmytriyeva O, Pankratova S, Owczarek S, Sonn K, Soroka V, Ridley CM, Marsolais A, Lopez-Hoyos M, Ambartsumian N, Lukanidin E, Bock E, Berezin V, Kiryushko D (2012). The metastasis-promoting S100A4 protein confers neuroprotection in brain injury. Nat Commun.

[39] Hibino T, Sakaguchi M, Miyamoto S, Yamamoto M, Motoyama A, Hosoi J, Shimokata T, Ito T, Tsuboi R, Huh NH (2013). S100A9 is a novel ligand of EMMPRIN that promotes melanoma metastasis. Cancer Res.

[40] Hankins JL, Ward KE, Linton SS, Barth BM, Stahelin RV, Fox TE, Kester M (2013). Ceramide 1-phosphate mediates endothelial cell invasion via the annexin a2-p11 heterotetrameric protein complex. J Biol Chem.

[41] Donato R (2003). Intracellular and extracellular roles of S100 proteins. Microsc Res Tech.

[42] Gendler SJ, Cohen EP, Craston A, Duhig T, Johnstone G, Barnes D (1990). The locus of the polymorphic epithelial mucin (PEM) tumour antigen on chromosome 1q21 shows a high frequency of alteration in primary human breast tumours. Int J Cancer.

[43] Weterman MA, Wilbrink M, Dijkhuizen T, van den Berg E, Geurts van Kessel A (1996). Fine mapping of the 1q21 breakpoint of the papillary renal cell carcinoma-associated (X;1) translocation. Hum Genet.

[44] Ohuchida K, Mizumoto K, Miyasaka Y, Yu J, Cui L, Yamaguchi H, Toma H, Takahata S, Sato N, Nagai E, Yamaguchi K, Tsuneyoshi M, Tanaka M (2007). Over-expression of S100A2 in pancreatic cancer correlates with progression and poor prognosis. J Pathol.

[45] Gupta S, Hussain T, MacLennan GT, Fu P, Patel J, Mukhtar H (2003). Differential expression of S100A2 and S100A4 during progression of human prostate adenocarcinoma. J Clin Oncol.

[46] Andersen K, Nesland JM, Holm R, Flørenes VA, Fodstad Ø, Maelandsmo GM (2004). Expression of S100A4 combined with reduced E-cadherin expression predicts patient outcome in malignant melanoma. Mod Pathol.

[47] Zou M, Al-Baradie RS, Al-Hindi H, Farid NR, Shi Y (2005). S100A4 (Mts1) gene overexpression is associated with invasion and metastasis of papillary thyroid carcinoma. Br J Cancer.

[48] Zhou G, Xie TX, Zhao M, Jasser SA, Younes MN, Sano D, Lin J, Kupferman ME, Santillan AA, Patel V, Gutkind JS, Ei-Naggar AK, Emberley ED (2008). Reciprocal negative regulation between S100A7/psoriasin and β-catenin signaling plays an important role in tumor progression of squamous cell carcinoma of oral cavity. Oncogene.

[49] Bulk E, Sargin B, Krug U, Hascher A, Jun Y, Knop M, Kerkhoff C, Gerke V, Liersch R, Mesters RM, Hotfilder M, Marra A, Koschmieder S (2009). S100A2 induces metastasis in non–small cell lung cancer. Clin Cancer Res.

[50] Naz S, Bashir M, Ranganathan P, Bodapati P, Santosh V, Kondaiah P (2014). Protumorigenic actions of S100A2 involve regulation of PI3/Akt signaling and functional interaction with Smad3. Carcinogenesis.

[51] Salama I, Malone PS, Mihaimeed F, Jones JL (2008). A review of the S100 proteins in cancer. Eur J Surg Oncol.

[52] Adam PJ, Boyd R, Tyson KL, Fletcher GC, Stamps A, Hudson L, Poyser HR, Redpath N, Griffiths M, Steers G, Harris AL, Patel S, Berry J (2003). Comprehensive proteomic analysis of breast cancer cell membranes reveals unique proteins with potential roles in clinical cancer. J Biol Chem.

[53] Jin Q, Chen H, Luo A, Ding F, Liu Z (2011). S100A14 stimulates cell proliferation and induces cell apoptosis at different concentrations via receptor for advanced glycation end products (RAGE). PLoS One.

[54] Xu C, Chen H, Wang X, Gao J, Che Y, Li Y, Ding F, Luo A, Zhang S, Liu Z (2014). S100A14, a member of the EF-hand calcium-binding proteins, is overexpressed in breast cancer and acts as a modulator of HER2 signaling. J Biol Chem.

[55] Chen H, Yuan Y, Zhang C, Luo A, Ding F, Ma J, Yang S, Tian Y, Tong T, Zhan Q, Liu Z (2012). Involvement of S100A14 protein in cell invasion by affecting expression and function of matrix metalloproteinase (MMP)-2 via p53-dependent transcriptional regulation. J Biol Chem.

[56] Uhlén M, Björling E, Agaton C, Szigyarto CA, Amini B, Andersen E, Andersson AC, Angelidou P, Asplund A, Asplund C, Berglund L, Bergström K, Brumer H (2005). A human protein atlas for normal and cancer tissues based on antibody proteomics. Mol Cell Proteomics.

[57] Uhlén M, Fagerberg L, Hallström BM, Lindskog C, Oksvold P, Mardinoglu A, Sivertsson Å, Kampf C, Sjöstedt E, Asplund A, Olsson I, Edlund K, Lundberg E (2015). Proteomics. Tissue-based map of the human proteome. Science.

[58] GTEx Consortium (2015). Human genomics. The Genotype-Tissue Expression (GTEx) pilot analysis: multitissue gene regulation in humans. Science.

[59] Takahashi H, Lassmann T, Murata M, Carninci P (2012). 5′ end–centered expression profiling using cap-analysis gene expression and next-generation sequencing. Nat Protoc.

[60] Yu NY, Hallström BM, Fagerberg L, Ponten F, Kawaji H, Carninci P, Forrest AR, Hayashizaki Y, Uhlén M, Daub CO, Fantom Consortium (2015). Complementing tissue characterization by integrating transcriptome profiling from the Human Protein Atlas and from the FANTOM5 consortium. Nucleic Acids Res.

[61] Döbróssy L (2005). Epidemiology of head and neck cancer: magnitude of the problem. Cancer Metastasis Rev.

[62] Johnson N (2001). Tobacco use and oral cancer: a global perspective. J Dent Educ.

[63] Massano J, Regateiro FS, Januário G, Ferreira A (2006). Oral squamous cell carcinoma: review of prognostic and predictive factors. Oral Surg Oral Med Oral Pathol Oral Radiol Endod.

[64] Lunde ML, Roman E, Warnakulasuriya S, Mehrotra R, Laranne J, Vasstrand EN, Ibrahim SO (2014). Profiling of chromosomal changes in potentially malignant and malignant oral mucosal lesions from South and South-East Asia using array-comparative genomic hybridization. Cancer Genomics Proteomics.

[65] Roman E, Meza-Zepeda LA, Kresse SH, Myklebost O, Vasstrand EN, Ibrahim SO (2008). Chromosomal aberrations in head and neck squamous cell carcinomas in Norwegian and Sudanese populations by array comparative genomic hybridization. Oncol Rep.

[66] Sapkota D, Bruland O, Bøe OE, Bakeer H, Elgindi OA, Vasstrand EN, Ibrahim SO (2008). Expression profile of the S100 gene family members in oral squamous cell carcinomas. J Oral Pathol Med.

[67] Lee SW, Tomasetto C, Swisshelm K, Keyomarsi K, Sager R (1992). Down-regulation of a member of the S100 gene family in mammary carcinoma cells and reexpression by azadeoxycytidine treatment. Proc Natl Acad Sci U S A.

[68] Tulchinsky E, Grigorian M, Tkatch T, Georgiev G, Lukanidin E (1995). Transcriptional regulation of the mts1 gene in human lymphoma cells: the role of DNA-methylation. Biochim Biophys Acta.

[69] Lindsey JC, Lusher ME, Anderton JA, Gilbertson RJ, Ellison DW, Clifford SC (2007). Epigenetic deregulation of multiple S100 gene family members by differential hypomethylation and hypermethylation events in medulloblastoma. Br J Cancer.

[70] Leśniak W (2011). Epigenetic regulation of S100 protein expression. Clin Epigenetics.

[71] Ji J, Zhao L, Wang X, Zhou C, Ding F, Su L, Zhang C, Mao X, Wu M, Liu Z (2004). Differential expression of S100 gene family in human esophageal squamous cell carcinoma. J Cancer Res Clin Oncol.

[72] Chen H, Yu D, Luo A, Tan W, Zhang C, Zhao D, Yang M, Liu J, Lin D, Liu Z (2009). Functional role of S100A14 genetic variants and their association with esophageal squamous cell carcinoma. Cancer Res.

[73] Chen H, Ma J, Sunkel B, Luo A, Ding F, Li Y, He H, Zhang S, Xu C, Jin Q, Wang Q, Liu Z (2013). S100A14: novel modulator of terminal differentiation in esophageal cancer. Mol Cancer Res.

[74] Zhu M, Wang H, Cui J, Li W, An G, Pan Y, Zhang Q, Xing R, Lu Y (2017). Calcium-binding protein S100A14 induces differentiation and suppresses metastasis in gastric cancer. Cell Death Dis.

[75] Zhang Q, Zhu M, Cheng W, Xing R, Li W, Zhao M, Xu L, Li E, Luo G, Lu Y (2015). Downregulation of 425G>a variant of calcium-binding protein S100A14 associated with poor differentiation and prognosis in gastric cancer. J Cancer Res Clin Oncol.

[76] Kim G, Chung JY, Jun SY, Eom DW, Bae YK, Jang KT, Kim J, Yu E, Hong SM (2013). Loss of S100A14 expression is associated with the progression of adenocarcinomas of the small intestine. Pathobiology.

[77] Wang HY, Zhang JY, Cui JT, Tan XH, Li WM, Gu J, Lu YY (2010). Expression status of S100A14 and S100A4 correlates with metastatic potential and clinical outcome in colorectal cancer after surgery. Oncol Rep.

[78] Cho H, Shin HY, Kim S, Kim JS, Chung JY, Chung EJ, Chun KH, Hewitt SM, Kim JH (2014). The role of S100A14 in epithelial ovarian tumors. Oncotarget.

[79] Qian J, Ding F, Luo A, Liu Z, Cui Z (2016). Overexpression of S100A14 in human serous ovarian carcinoma. Oncol Lett.

[80] Yao R, Lopez-Beltran A, Maclennan GT, Montironi R, Eble JN, Cheng L (2007). Expression of S100 protein family members in the pathogenesis of bladder tumors. Anticancer Res.

[81] Tanaka M, Ichikawa-Tomikawa N, Shishito N, Nishiura K, Miura T, Hozumi A, Chiba H, Yoshida S, Ohtake T, Sugino T (2015). Co-expression of S100A14 and S100A16 correlates with a poor prognosis in human breast cancer and promotes cancer cell invasion. BMC Cancer.

[82] Zhao FT, Jia ZS, Yang Q, Song L, Jiang XJ (2013). S100A14 promotes the growth and metastasis of hepatocellular carcinoma. Asian Pac J Cancer Prev.

[83] Ding F, Wang D, Li XK, Yang L, Liu HY, Cui W, Liu ZH, Che YQ (2018). Overexpression of S100A14 contributes to malignant progression and predicts poor prognosis of lung adenocarcinoma. Thorac Cancer.

[84] Katono K, Sato Y, Kobayashi M, Saito K, Nagashio R, Ryuge S, Igawa S, Nakashima H, Shiomi K, Satoh Y, Ichinoe M, Murakumo Y, Saegusa M, Masuda N (2017). Clinicopathological significance of S100A14 expression in lung adenocarcinoma. Oncol Res Treat.

[85] Zhang X, Li W, Hou Y, Niu Z, Zhong Y, Zhang Y, Yang S (2014). Comparative membrane proteomic analysis between lung adenocarcinoma and normal tissue by iTRAQ labeling mass spectrometry. Am J Transl Res.

[86] el-Deiry WS, Tokino T, Velculescu VE, Levy DB, Parsons R, Trent JM, Lin D, Mercer WE, Kinzler KW, Vogelstein B (1993). WAF1, a potential mediator of p53 tumor suppression. Cell.

[87] Martinsson H, Yhr M, Enerbäck C (2005). Expression patterns of S100A7 (psoriasin) and S100A9 (calgranulin-B) in keratinocyte differentiation. Exp Dermatol.

[88] Wolf R, Lewerenz V, Büchau AS, Walz M, Ruzicka T (2007). Human S100A15 splice variants are differentially expressed in inflammatory skin diseases and regulated through Th1 cytokines and calcium. Exp Dermatol.

[89] Sapkota D, Bruland O, Parajuli H, Osman TA, Teh MT, Johannessen AC, Costea DE (2015). S100A16 promotes differentiation and contributes to a less aggressive tumor phenotype in oral squamous cell carcinoma. BMC Cancer.

[90] Zhao Y, Yao F, Tang W, Gu H, Zhao H (2017). *S100A14* rs11548103 G>A polymorphism is associated with a decreased risk of esophageal cancer in a Chinese population. Oncotarget.

[91] Tan M, Heizmann CW, Guan K, Schafer BW, Sun Y (1999). Transcriptional activation of the human S100A2 promoter by wild-type p53. FEBS Lett.

[92] Zhao H, Guo E, Hu T, Sun Q, Wu J, Lin X, Luo D, Sun C, Wang C, Zhou B, Li N, Xia M, Lu H (2016). KCNN4 and S100A14 act as predictors of recurrence in optimally debulked patients with serous ovarian cancer. Oncotarget.

[93] Wang X, Yang J, Qian J, Liu Z, Chen H, Cui Z (2015). S100A14, a mediator of epithelial-mesenchymal transition, regulates proliferation, migration and invasion of human cervical cancer cells. Am J Cancer Res.

[94] Bertini I, Borsi V, Cerofolini L, Das Gupta S, Fragai M, Luchinat C (2013). Solution structure and dynamics of human S100A14. J Biol Inorg Chem.

[95] He H, Li S, Chen H, Li L, Xu C, Ding F, Zhan Y, Ma J, Zhang S, Shi Y, Qu C, Liu Z (2014). 12-O-tetradecanoylphorbol-13-acetate promotes breast cancer cell motility by increasing S100A14 level in a Kruppel-like transcription factor 4 (KLF4)-dependent manner. J Biol Chem.

[96] McKiernan E, McDermott EW, Evoy D, Crown J, Duffy MJ (2011). The role of S100 genes in breast cancer progression. Tumour Biol.

[97] Ko CH, Cheng CF, Lai CP, Tzu TH, Chiu CW, Lin MW, Wu SY, Sun CY, Tseng HW, Wang CC, Kuo ZK, Wang LM, Chen SF (2013). Differential proteomic analysis of cancer stem cell properties in hepatocellular carcinomas by isobaric tag labeling and mass spectrometry. J Proteome Res.

[98] Massard C, Deutsch E, Soria JC (2006). Tumour stem cell-targeted treatment: elimination or differentiation. Ann Oncol.

[99] Ehmsen S, Hansen LT, Bak M, Brasch-Andersen C, Ditzel HJ, Leth-Larsen R (2015). S100A14 is a novel independent prognostic biomarker in the triple-negative breast cancer subtype. Int J Cancer.

